# Supplementary material for: Angiogenesis Driven by the CEBPD–hsa-miR-429–VEGFA Signaling Axis Promotes Urothelial Carcinoma Progression
Source: Cells. 2022 Feb 11;11(4):638. doi: 10.3390/cells11040638 (PMC8870255; doi:10.3390/cells11040638)
Supplement: Supplementary file 1 [file cells-11-00638-s001.zip › cells-1579825-supplementary.pdf]

Table S1**.** VEGFA is a potentially predicted target for hsa-miR-429 by Miranda database (http://cbio.mskcc.org/miRNA2003/miranda.html) described as our previous research paper [1].

	miRNA targets*	
	Validated targets	Predicted targets
hsa-miR-429	WASF3|XIAP|MYC|ZEB1|OSTF1|BCL2|ZFPM2|SOX2|RERE|ZEB2	ERMP1|ATP2B1|CYP1B1|SCD|MRAS|LPAR1|AFF1|TMTC1|CHST11|LFNG|SDC2|FBXO32|FBN2|TNFSF9|EGR1|CD70|ZNF281|**VEGFA**|TREM1|CEBPD|TGFBR1|EIF5A2|OGFRL1|OMA1|TIMP2|REXO1|HK2|CBX5|KLF4|IFNGR2|ZBTB20|JUN|OSMR|ID2

1. Chan, T.-C.; Chen, Y.-T.; Tan, K.T.; Wu, C.-L.; Wu, W.-J.; Li, W.-M.; Wang, J.-M.; Shiue, Y.-L.; Li, C.-F. Biological significance of MYC and CEBPD coamplification in urothelial carcinoma: Multilayered genomic, transcriptional and posttranscriptional positive feedback loops enhance oncogenic glycolysis. *Clinical and Translational Medicine* **2021**, *11*, e674, doi:10.1002/ctm2.674.
